# SWEET Transporters and the Potential Functions of These Sequences in Tea (*Camellia sinensis*)

**DOI:** 10.3389/fgene.2021.655843

**Published:** 2021-03-31

**Authors:** Lan Jiang, Cheng Song, Xi Zhu, Jianke Yang

**Affiliations:** ^1^Central Laboratory, Yijishan Hospital of Wannan Medical College, Wuhu, China; ^2^Key Laboratory of Non-coding RNA Transformation Research of Anhui Higher Education Institution, Yijishan Hospital of Wannan Medical College, Wuhu, China; ^3^College of Biological and Pharmaceutical Engineering, West Anhui University, Luan, China; ^4^Department of Medicine III, University Hospital, LMU Munich, Munich, Germany; ^5^School of Preclinical Medicine, Wannan Medical College, Wuhu, China

**Keywords:** *SWEET*, duplication, *Camellia sinensis*, expression, microsynteny

## Abstract

Tea (*Camellia sinensis*) is an important economic beverage crop. Its flowers and leaves could be used as healthcare tea for its medicinal value. SWEET proteins were recently identified in plants as sugar transporters, which participate in diverse physiological processes, including pathogen nutrition, seed filling, nectar secretion, and phloem loading. Although *SWEET* genes have been characterized and identified in model plants, such as *Arabidopsis thaliana* and *Oryza sativa*, there is very little knowledge of these genes in *C. sinensis*. In this study, 28 *CsSWEET*s were identified in *C. sinensis* and further phylogenetically divided into four subfamilies with *A. thaliana*. These identified *CsSWEET*s contained seven transmembrane helixes (TMHs) which were generated by an ancestral three-TMH unit with an internal duplication experience. Microsynteny analysis revealed that the large-scale duplication events were the main driving forces for members from *CsSWEET* family expansion in *C. sinensis*. The expression profiles of the 28 *CsSWEET*s revealed that some genes were highly expressed in reproductive tissues. Among them, *CsSWEET1a* might play crucial roles in the efflux of sucrose, and *CsSWEET17b* could control fructose content as a hexose transporter in *C. sinensis*. Remarkably, *CsSWEET12* and *CsSWEET17c* were specifically expressed in flowers, indicating that these two genes might be involved in sugar transport during flower development. The expression patterns of all *CsSWEET*s were differentially regulated under cold and drought treatments. This work provided a systematic understanding of the members from the *CsSWEET* gene family, which would be helpful for further functional studies of *CsSWEET*s in *C. sinensis*.

## Introduction

SWEET is a novel kind of low-affinity sugar transporter, which does not depend on the pH value of the environment and transports in two directions along the concentration gradient (Yuan and Wang, 2013). The members of the *SWEET* family are widely distributed, which are ubiquitous in higher eukaryotes and also distributed in protozoa, metazoa, fungi, bacteria, and archaea (Hamada et al., 2005; Saier et al., 2006; Xuan et al., 2013). The membrane proteins encoded by the *SWEET* have a certain number of conserved transmembrane domains, which are named MtN3/saliva (Hamada et al., 2005). This domain was first found in nodulin from the roots of *Medicago sativa* (Gamas et al., 1996). Recently, phylogenetic analysis has been performed for the main protein members of the SWEET family, which are divided into three branches: (i) the plant-like SWEET, most of which contain two MtN3/saliva transmembrane domains, (ii) the animal-like SWEET, which has two specific MtN3/saliva domains, and (iii) some SWEET proteins from bacteria to Archaea (cocci) and nematodes, which consist of an MtN3/saliva domain containing three transmembrane alpha helices (Yuan and Wang, 2013). A large number of *SWEET* family members have been found in *Arabidopsis thaliana* (Wipf et al., 2020), *Vitis vinifera* (Chong et al., 2014), *Solanum lycopersicum* (Shammai et al., 2018), *Manihot esculenta* (Cao et al., 2019), *Eucalyptus grandis* (Yin et al., 2020), etc., and are involved in many physiological processes. For instance, *RPG1* (*ATSWEET8*) plays a vital role in microspore outer wall formation in *Arabidopsis* (Guan et al., 2008); in *Oryza sativa*, the *SWEET* family members are also involved in reproductive development (Wang et al., 2010); *SAG29* (*AtSWEETl5*), located on the plasma membrane in *Arabidopsis*, can regulate cell activity in a hypersaline environment (Seo et al., 2011). The *SWEET*s also participate in the regulation of the aging process. The overexpression of *OsSWEET5* can cause growth delay and premature aging in rice seedlings, while no phenotypic changes were observed in the knockout ones (Zhou et al., 2014). Chu et al. (2006) found that the rice harboring mutant *OsSWEET11* (*Xa13*) can resist powdery mildew, suggesting that *SWEET*s not only served as a glucose transporter but also participated in the interaction between host and pathogen. However, the members of the *CsSWEET* gene family have not yet been identified, and the roles of *CsSWEET*s remain unclear in tea plant (*Camellia sinensis*).

*Camellia sinensis*, which originated from the tropical area of Southwest China, is an important economic beverage crop in China (Wei et al., 2018; Zhang et al., 2020b). Sugar transport and homeostasis contribute to plant growth and development (May et al., 1998; Lastdrager et al., 2014; Rahimi et al., 2019; Pan et al., 2020; Saddhe et al., 2020). Some published papers focused on the genes that participated in sugar metabolism, such as hexose kinase, invertase, and galactinol synthase, in *C. sinensis* (Yue et al., 2015; Zhou et al., 2017; Samarina et al., 2020). Due to the economic interest in *C. sinensis* as a beverage crop, its genome, proteome, and transcriptome were recently sequenced and released (Wei et al., 2018; Wu et al., 2018; Liu et al., 2020; Xia et al., 2020; Zhang et al., 2020b), which help us to analyze the *SWEET* genes in *C. sinensis* systematically. In the present study, 26 *CsSWEET*s in *C. sinensis* were identified, and their gene structures, phylogenetic, microsynteny, and expression were analyzed. Our study revealed the functions of these *CsSWEET*s and provided candidate genes for further research.

## Materials and Methods

### Database Search for *C. sinensis* Genome

The *C. sinensis* genome with GFF annotation, CDS, and protein files were downloaded from the Tea Plant Information Archive^1^ (TPIA) database (Xia et al., 2019). The *AtSWEET*s of *A. thaliana* and *HsSWEET1* of *Homo sapiens* were obtained from TAIR^2^ and Genbank^3^, respectively. The HMM profile of MtN3/saliva domain (PF03083) was obtained from the Pfam database^4^ (Mistry et al., 2020), and the HMMER (version 3.1) software (Mistry et al., 2013) was used to identify CsSWEET proteins in *C. sinensis* genome (*E*-value ≤ 1e−3). The Pfam (version 33.1), SMART (version 9), and InterPro (version 5) were used to confirm the above-mentioned identified sequences with *E*-value ≤ 1e−3 (Jones et al., 2014; Letunic and Bork, 2018; Mistry et al., 2020). Finally, we manually discarded these sequences with a lack of complete or core MtN3/saliva domain for further analyses.

### Phylogenetic Analyses

The MAFFT (version 7) software was used to execute the multiple alignments of all SWEET proteins with default parameters (Katoh et al., 2005). The best substitution model of these SWEET proteins was determined by ModelFinder software (Kalyaanamoorthy et al., 2017). The maximum likelihood (ML) tree was determined by IQ-tree (version 2.1.2) software (Nguyen et al., 2015) with an SH-aLRT test for 1,000 random addition replicates and a bootstrap test for 10,000 replicates as described by Cao et al. (2020a). The Figtree software was used to visualize this ML tree.

### Internal Repeats and Topological Analyses

The ClustalX (version 2) software (Thompson et al., 2003) was used to create the multiple alignments of SWEETs from *C. sinensis* and *A. thaliana*. The HHrepID was used to identify the internal repeats in SWEET proteins (Söding et al., 2006). In addition, we also used the AveHAS to calculate the topological conservation (Zhai and Saier, 2001a,b) as well as create the hydropathy, amphipathicity, and similarity plots, respectively (Zhai and Saier, 2001a). The GFF annotation file was obtained from the TPIA (see text footnote 1) database (Xia et al., 2019). The TBtools (version 1) software was used to generate this gene structure map based on the GFF annotation file (Chen et al., 2020). The MEME online tool was used to predict the conserved motifs (Bailey et al., 2015).

### Microsynteny Analysis

The MicroSyn (version 1) software was used to detect the microsynteny of *CsSWEET* genes in *C. sinensis* genome with a threshold *E*-value of <1e−5 (Cai et al., 2011). In this study, we determined a syntenic block, a region which include three or more conserved homolog genes that were distributed within 15 genes upstream and downstream of *CsSWEET* genes as described by Cao et al. (2020b).

### RNA-Seq Expression Analysis

In our study, transcriptome data from various tissues in the public database NCBI contained three biological replicates. The RNA-seq reads, including seed, flower, stem, root, two and a bud, one and a bud, old leaf, mature leaf, the second leaf, the first leaf, lateral bud, apical bud, early stage lateral bud, CP24 (24 h after pollination; CP, cross-pollinated), CP48, CP72, SP24 (SP, self-pollinated), SP48, and SP72, were obtained from NCBI (PRJNA291116 and PRJNA230752). The Trimmomatic (version 0.33) was used to carried out the quality-based trimming (Bolger et al., 2014). The HISAT2 was used to map the paired reads to the *C. sinensis* genome with default parameters (Kim et al., 2019). The StringTie was used to calculate the fragments per kilobase of exon model per million reads mapped values of differently expressed genes (Pertea et al., 2016).

## Results and Discussion

### The Phylogenetic Analysis Divides *CsSWEET* Genes Into Four Subfamilies in *C. sinensis*

In the present study, we identified 26 *CsSWEET* genes in *C. sinensis* genome by using HMMER 3.0 software, similarly as in *Manihot esculenta*, *Jatropha curcas*, *Ricinus communis*, *Vernicia fordii*, *Malus* × *domestica*, *Oryza sativa*, *Zea mays*, *Glycine max*, and *Pyrus bretschneideri* (Chen et al., 2010; Yuan and Wang, 2013; Chong et al., 2014; Cao et al., 2019; Yin et al., 2020). Subsequently, we renamed the newly identified *CsSWEET*s based on the nomenclature of the *AtSWEET*s of *A. thaliana*. The detailed information of each *CsSWEET*, including chromosome position, gene identifiers, isoelectric point, molecular weight, and gene name, is shown in Table 1.

**TABLE 1:** Detailed information of SWEET family members in *C. sinensis*.

Gene name	Gene identifiers	Chromosome	Forward (+)/reverse (−)	3′ end	5′ end	p*I*	MW
*CsSWEET1a*	CSS0018035.1	Chr4	−	47,319,514	47,322,176	9.64	27,695.99
*CsSWEET1b*	CSS0018347.1	Chr9	−	71,038,986	71,045,420	9.68	26,377.41
*CsSWEET2a*	CSS0040201.1	Contig440	−	7,089	14,988	8.84	21,873.99
*CsSWEET2b*	CSS0003324.2	Chr5	−	46,529,212	46,534,196	8.81	25,966.73
*CsSWEET2c*	CSS0042514.1	Chr8	+	68,320,865	68,324,641	9.38	23,055.65
*CsSWEET3a*	CSS0003069.1	Chr7	+	174,818,978	174,820,449	8.61	25,941.05
*CsSWEET3b*	CSS0039909.1	Contig86	−	502,517	504,121	9.22	30,808.87
*CsSWEET3c*	CSS0028613.1	Chr1	+	98,968,341	98,969,945	9.22	30,736.72
*CsSWEET5a*	CSS0040324.1	Chr15	−	79,558,505	79,562,983	9.21	26,678.06
*CsSWEET5b*	CSS0037258.1	Chr2	+	6,354,873	6,363,319	8.54	51,845.3
*CsSWEET5c*	CSS0009453.1	Chr2	–	83,321,970	83,325,289	6.72	27,143.31
*CsSWEET7a*	CSS0014422.1	Chr1	+	220,693,547	220,710,933	5.25	80,809.52
*CsSWEET7b*	CSS0001069.1	Chr12	−	158,359,825	158,365,331	8.93	28,302.76
*CsSWEET7c*	CSS0033641.1	Chr12	−	158,634,675	158,640,280	9.06	28,329.83
*CsSWEET9a*	CSS0020524.1	Chr11	−	96,099,923	96,104,109	6.81	27,224.46
*CsSWEET9b*	CSS0016012.1	Chr11	−	96,128,414	96,130,178	8.72	30,791.65
*CsSWEET10a*	CSS0014835.1	Chr11	+	13,881,045	13,882,601	9.26	31,961.51
*CsSWEET10b*	CSS0011593.1	Chr15	−	49,947,912	49,948,754	7.66	21,250.28
*CsSWEET10c*	CSS0026264.1	Chr2	−	97,204,012	97,206,001	9	36,861.14
*CsSWEET12*	CSS0010858.1	Chr15	−	49,282,933	49,284,143	8.97	28,258.6
*CsSWEET15a*	CSS0049101.1	Chr6	+	3,037,473	3,039,942	7.75	30,227.9
*CsSWEET15b*	CSS0007875.1	Chr11	+	96,022,451	96,035,117	9.27	32,181.35
*CsSWEET15c*	CSS0017308.1	Chr11	+	96,051,665	96,053,111	9.5	26,881.44
*CsSWEET17a*	CSS0009124.1	Chr3	+	61,755,153	61,761,768	8.73	33,441.32
*CsSWEET17b*	CSS0021289.1	Chr9	−	8,102,406	8,106,125	6.19	26,228.91
*CsSWEET17c*	CSS0005451.1	Chr3	+	61,588,229	61,597,620	8.89	22,789.63

**FIGURE 1:**
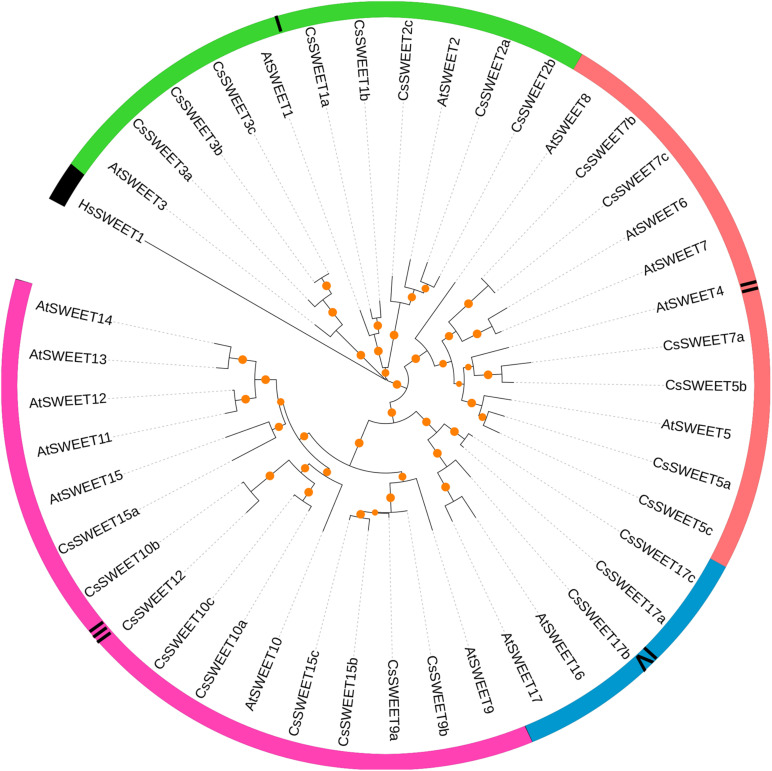
The phylogenetic relationships of *SWEET* genes in *Camellia sinensis*, *Arabidopsis thaliana*, and *Homo sapiens*. The maximum likelihood tree was built by IQ-tree software with bootstrap test (10,000 replicates).

To gain insight into the evolutionary relationship of *CsSWEET*s in *C. sinensis*, the *SWEET*s from *C. sinensis* and *A. thaliana* were aligned by MAFFT software. Remarkably, *HsSWEET1* from *Homo sapiens* was an outgroup, which has only one copy and could transport glucose in *H. sapiens*. In *V. fordii*, Cao et al. (2019) found that *VfSWEET1* contributes to the balance of sucrose levels, and other *VfSWEET*s may play key roles in the growth and development of plants (Cao et al., 2019). In *A. thaliana*, *AtSWEET*s were identified with versatile functions, such as control fructose content and sucrose efflux (Chen et al., 2010, 2012). In our study, the *SWEET*s from *C. sinensis* and *A. thaliana* were clustered into four subfamilies, including subfamily I, subfamily II, subfamily III, and subfamily IV (Figure 1). Subsequently, there was at least one *AtSWEET* from *A. thaliana* in each subfamily. Previously published papers have confirmed that members from different *SWEET* subfamilies may have multiple biological functions, such that the members from subfamily I and subfamily II were efficient hexose transporters (Figure 1), the members from subfamily III may be responsible for sucrose transporters, and the members from subfamily IV appear to be predominantly fructose transporters (Chen et al., 2010, 2012; Hu et al., 2018; Cao et al., 2019).

### The Structure Analyses Reveal the Distribution of Exon–Intron and the Conserved Composition of Motif in *C. sinensis*

According to the predicted sequences and GFF annotation files, we determined the distribution of exon–intron of the 26 *CsSWEET* genes in *C. sinensis*. As shown in Supplementary Figure 1, most *CsSWEET* genes contained five introns; however, several of the members from the *CsSWEET* gene family contained two, three, or four introns. Remarkably, there are extreme differences in the number of introns from the *CsSWEET* subfamily II, ranging from 4 to 14, indicating that these *CsSWEET*s may have experienced intron loss or gain some during evolution (Supplementary Figure 1). These results were also confirmed by previous studies. For example, Cao et al. (2019) found that the number of introns from *VfSWEET*s ranged from four to 10. Additionally, we found that, although the lengths of members from *CsSWEET* gene family vary, introns stepped into almost the same position of the gene open reading frame.

The MEME website was used to identify the sequence characteristics of CsSWEET genes. Subsequently, we determined 20 distinct motifs for CsSWEETs and annotated these motifs by Pfam and SMART database (Letunic and Bork, 2018; Mistry et al., 2020). The SWEET domain (MtN3/saliva) was encoded by motifs 1, 2, 3, and 4 (Supplementary Figure 2). However, the remaining motifs were not scanned for function annotations in Pfam and SMART database. Overall, the CsSWEET proteins within the same subfamily exhibited similar conserved motifs, especially in paralogs gene pairs, suggesting that these sequences might contain similar functions in *C. sinensis*.

### Internal Repeat and Transmembrane Domain Analysis of the *CsSWEET*s Reveal Insight Into Key Functional Residues

**FIGURE 2:**
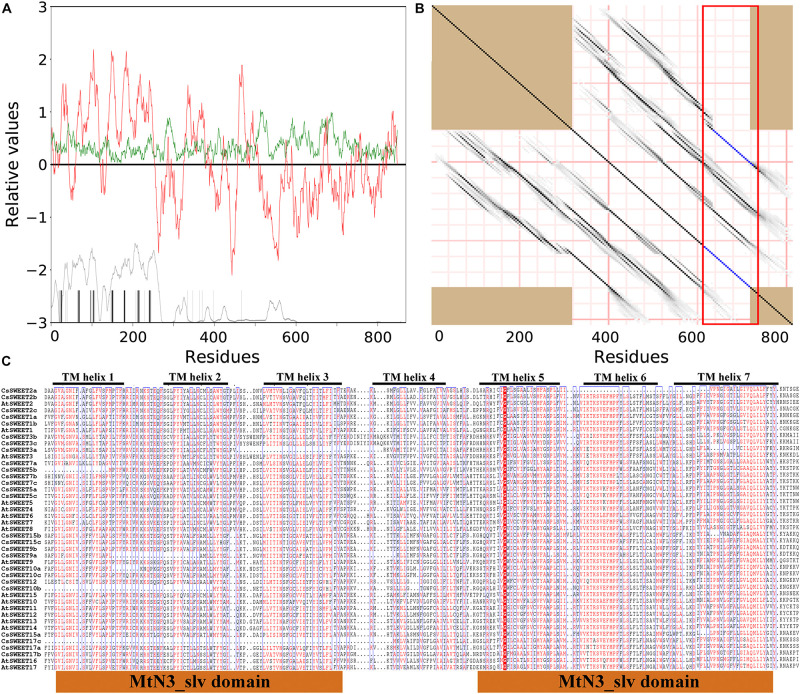
Sequence characteristics of SWEETs in *Camellia sinensis*. **(A)** The red upper lines and green upper lines indicate hydrophobicity and amphipathicity, respectively. **(B)** Internal repeat of SWEETs. Above the dark diagonal lines suggests the duplicated regions. **(C)** Alignment of SWEET sequences. The highly conserved residues are represented by boxes.

In general, SWEET proteins have been confirmed to contain seven transmembrane helixes (TMHs) in eukaryotes (Feng and Frommer, 2015; Jia et al., 2017). As expected, the membrane topology of CsSWEETs and AtSWEETs revealed that these proteins contained a moderately amphipathic nature with seven well-conserved hydrophobicity peaks (TMH1–TMH7) as determined by AveHAS plot (Figures 2A,B). Compared with TMHs, the loop area between two TMHs is not conservative in this study (Figure 2C). Previously published manuscripts have shown that the internal fusion and duplication of small fragments play an important role in the SWEETs evolution, which has been the focus of discussion by researchers (Hu et al., 2018; Cao et al., 2019). Researchers have studied the internal duplication of the SWEET proteins (Li et al., 2017; Hu et al., 2018; Cao et al., 2019), but direct evidence of the CsSWEETs from *C. sinensis* has not been reported. To confirm the presence of internal duplication, a repeat analysis of CsSWEETs and AtSWEETs was performed by the HHrepID program (Söding et al., 2006). The results confirmed that TMH1–TMH3 and TMH5–TMH7 were located in the duplicated regions, implying that SWEETs originate from an ancestral three-TMH unit which experienced an internal repeat duplication, which is in agreement with previously published papers (Li et al., 2017; Hu et al., 2018; Cao et al., 2019).

Li et al. (2017) revealed that Y, P, and D from TMH2, TMH5, and TMH7 were fully conserved residues in pear, respectively (Li et al., 2017). Among these three residues, Y mutation into A will lead to the loss of transport function in *A. thaliana* (Xuan et al., 2013). Proline (P) is a key component of TMHs and allows for dynamic processes during the transport cycle as described by Deber and Therien (2002). In the present study, we suggested that these three residues (Y, P, and D) might contain important functions within the activity of CsSWEETs in *C. sinensis*. In addition, the mutation of 58 amino acid residues G into D can significantly reduce AtSWEET1 activity in *A. thaliana* (Xuan et al., 2013). However, this amino acid is not completely conserved in *C. sinensis*, including the residue G in CsSWEET12 that had changed to N and in CsSWEET15a that had changed to K, but these proteins also have transport activity (Figure 2C), which has been verified by RNA-seq. We also noted that the first and the second MtN3/saliva domain included TMH1–TMH3 and TMH5–TMH7, respectively.

### Microsynteny Analysis Indicates That Large-Scale Duplication Events Contribute to the Expansion for *CsSWEET* Genes in *C. sinensis*

**FIGURE 3:**
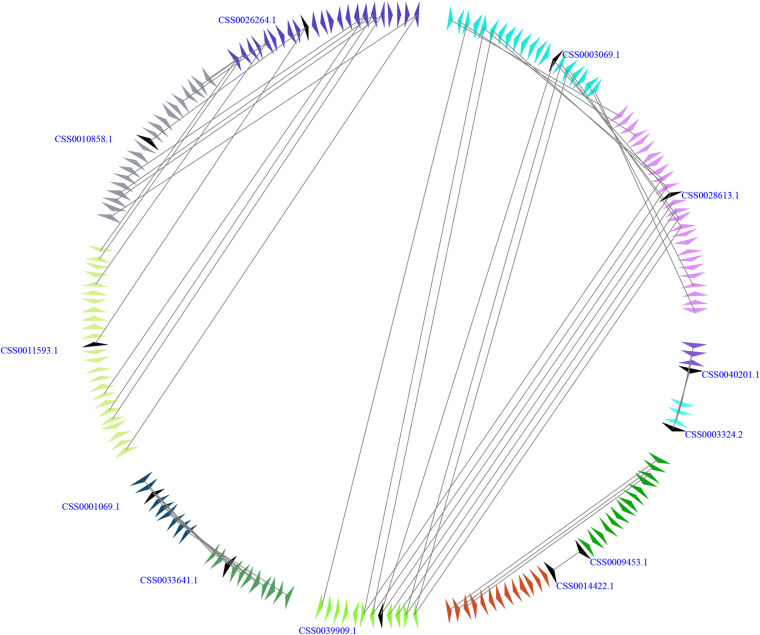
Microsynteny relationships of *SWEET* genes in *Camellia sinensis*. A series of triangles indicate genomic fragments and the genes’ orientations. The duplicated gene pairs on the two fragments are linked by gray lines.

The genome of *C. sinensis* experienced two rounds of whole-genome duplications (WGDs), including an ancient WGD event (∼90 to 100 Mya) and a recent WGD event (∼30 to 40 Mya) (Wei et al., 2018; Wu et al., 2018; Liu et al., 2020; Xia et al., 2020; Zhang et al., 2020b). To gain insight into the expansion mechanism of the members from *CsSWEET* gene family, we carried out a microsynteny analysis with *E*-value 10−5 as described by Cao et al. (2020b). In our study, *CsSWEET3c/CsSWEET3a*, *CsSWEET10c/CsSWEET12*, *CsSWEET10c/CsSWEET10b*, *CsSW EET7b/CsSWEET7c*, *CsSWEET3c/CsSWEET3b*, *CsSWEET3a/C sSWEET3b*, *CsSWEET7a/CsSWEET5c*, and *CsSWEET2b/Cs SWEET2a* contained more than three pairs of conserved flanking genes, suggesting that significant microsynteny might occur in the *CsSWEET* genes (Figure 3). These results indicated that the large-scale duplication events contribute to the expansion for *CsSWEET* genes in *C. sinensis*.

Generally, the evolutionary data of WGD events were estimated by *K*s values (Wang et al., 2011; Tiley et al., 2018; Zwaenepoel and Van De Peer, 2019). As shown in Table 1, we found that the *K*s values of *CsSWEET* duplication pairs ranged from 0.0103 to 2.1158 (Supplementary Table 1 and Supplementary Figure 3). Subsequently, we found that *CsSWEET3c/CsSWEET3a*, *CsSWEET3a/CsSWEET3b*, and *CsSWEET2b/CsSWEET2a* might be generated through a recent WGD event, while *CsSWEET3c/CsSWEET3b* and *CsSWEET10c/CsSWEET12* might be generated through an ancient WGD event, suggesting that these two rounds of WGD events might play key roles in the expansion for *CsSWEET* genes in *C. sinensis*.

### The Expression of *CsSWEET*s Reveals Possible Functions in *C. sinensis*

**FIGURE 4:**
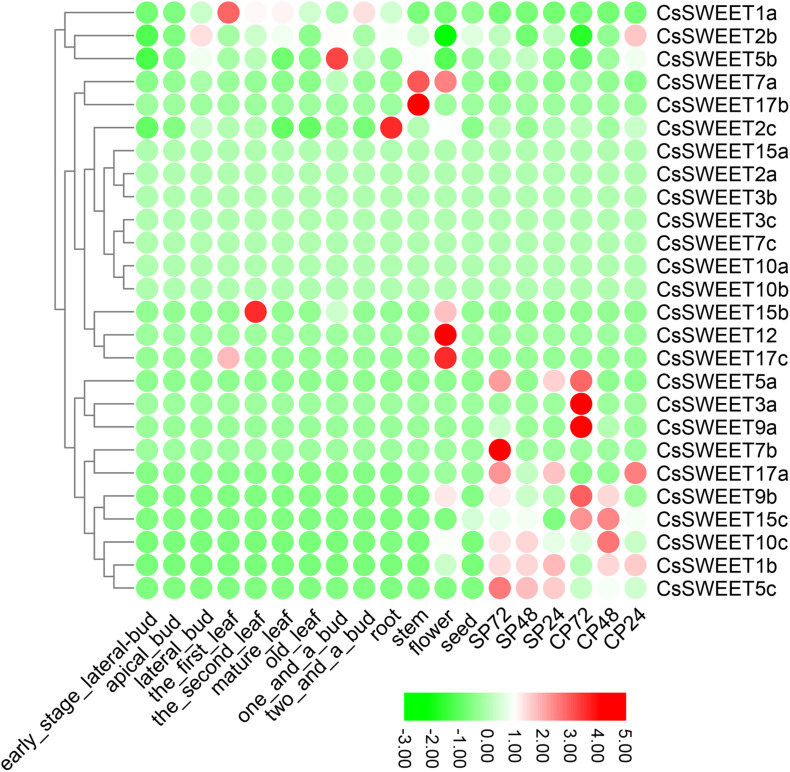
Expression profiles of the *CsSWEET*s in different tissues, including seed, flower, stem, root, two and a bud, one and a bud, old leaf, mature leaf, the second leaf, the first leaf, lateral bud, apical bud, and early stage lateral bud. CP24 = 24 h after pollination; CP, cross-pollinated – CP48, CP72; SP24 (SP, self-pollinated), SP48, and SP72.

Previously published work provided transcriptome data for *CsSWEET* genes in *C. sinensis* (Xia et al., 2019; Xia et al., 2020). The members from *SWEET* gene family are found to play diverse functional roles in various tissues and contribute to different sugar transport mechanisms in plants (Chen, 2014; Hedrich et al., 2015; Li et al., 2017; Cao et al., 2019). Differential expression analysis of *CsSWEET*s in *C. sinensis* is helpful for us to find out the specialized functions of these *CsSWEET*s in sugar transport from the practical application point of view. In the present study, we collected transcriptome data from 19 different tissues, including seed, flower, stem, root, two and a bud, one and a bud, old leaf, mature leaf, the second leaf, the first leaf, lateral bud, apical bud, early stage lateral bud, CP24 (24 h after pollination; CP, cross-pollinated), CP48, CP72, SP24 (SP, self-pollinated), SP48, and SP72. The published papers indicated that the expression of *SWEET*s in seeds pollen, flower, and embryo sacs in *Sorghum bicolor*, *V. fordii*, *Litchi chinensis*, *O. sativa*, and *A. thaliana* was higher (Yuan and Wang, 2013; Mizuno et al., 2016; Cao et al., 2019; Xie et al., 2019), implying that these genes may contribute to reproductive development. Here *CsSWEET1a* that was clustered into the same subfamily with *VfSWEET1* from *V. fordii*, *PbSWEET14* from *P. bretschneideri*, and *AtSWEET1* from *A. thaliana* was extremely highly expressed in the leaf of *C. sinensis*. *PbSWEET14* and *VfSWEET1* contribute to the efflux of sucrose in leaves (Li et al., 2017; Cao et al., 2019), while *AtSWEET1* plays an important role as a single glucose transporter in multiple systems. Therefore, the *CsSWEET1a* might play crucial roles in the efflux of sucrose and act as a glucose uniporter in the leaves of *C. sinensis* (Figure 4). *AtSWEET16* and *AtSWEET17* were shown to be vacuolar hexose transporters that controlled fructose content (Chen et al., 2010). The *CsSWEET17b*, which is the orthologous gene of *AtSWEET16* and *AtSWEET17*, was extremely highly expressed in the stems of *C. sinensis*, suggesting that this gene could control fructose content as a hexose transporter. The expression patterns of *CsSWEET2a*, *CsSWEET5b*, and *CsSWEET7a* were diverse in several tissues, implying that these *CsSWEET*s might play a role in these tested tissues of *C. sinensis* (Figure 4).

In *C. sinensis*, the expression of *CsSWEET*s was examined in pollen development. We found that 10 *CsSWEET*s, including *CsSWEET5a*, *CsSWEET3a*, *CsSWEET9a*, *CsSWEET7b*, *CsSWEET17a*, *CsSWEET9b*, *CsSWEET15c*, *CsSWEET10c*, *CsSWEET1b*, and *CsSWEET5c*, were highly expressed in one or several pollen stages of *C. sinensis* (Figure 4). As previously reported, Chen et al. (2012) identified *AtSWEET11* and *AtSWEET12* that contributed to phloem loading and pollen nutrition. Gao et al. (2018) found that the *OsSWEET11* from *O. sativa* can greatly affect the starch pollen content. In our study, *CsSWEET9b*, *CsSWEET9a*, and *CsSWEET10c* are the orthologous genes of *AtSWEET11* and *AtSWEET12*, indicating that these highly expressed genes may share similar roles and functions.

### Stress-Induced Expression Profiles of *CsSWEET*s in *C. sinensis*

**FIGURE 5:**
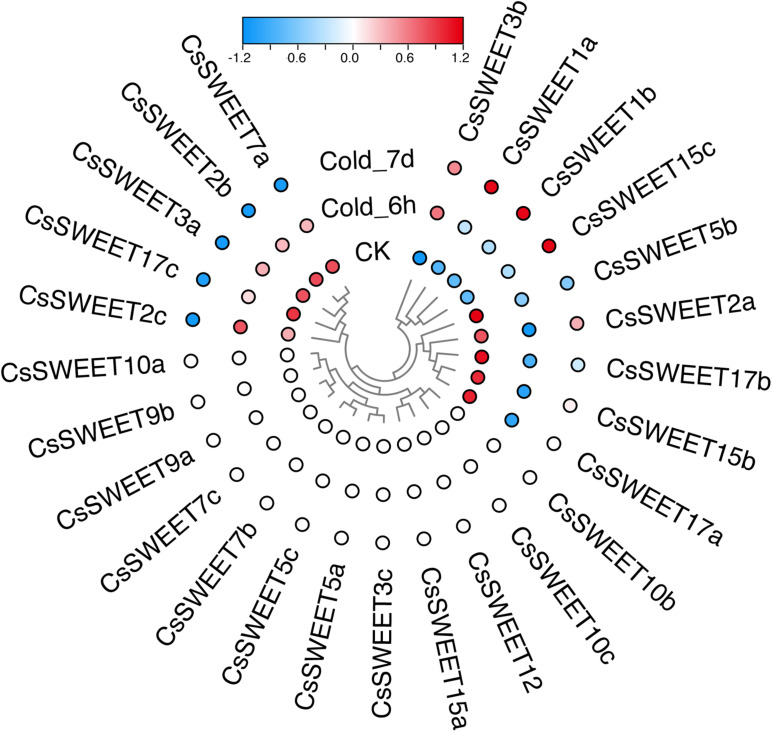
Expression profiles of the *CsSWEET*s in response to cold treatment.

**FIGURE 6:**
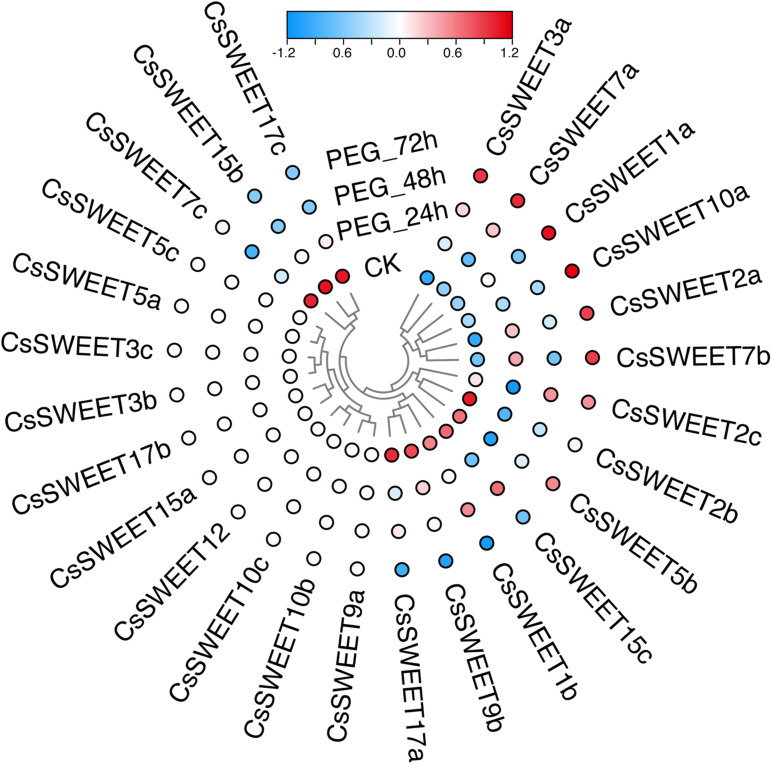
Expression profiles of the *CsSWEET*s in response to drought treatment.

Plants often suffer from a variety of abiotic stresses in the process of growth and development (Franco et al., 2011; Keunen et al., 2013; Etesami, 2018). Previous studies have shown that *SWEET*s help to control the responses to environmental stresses in plants (Li et al., 2018; Zhang et al., 2020a). Therefore, the expression patterns of *CsSWEET*s were investigated in response to different stresses, including cold stress and drought stress. In our study, the RNA-seq data for cold stress and drought stress were obtained from the TPIA (see text footnote 1) database (Xia et al., 2019). In response to drought, seven genes (*CsSWEET3a*, *CsSWEET7a*, *CsSWEET1a*, *CsSWEET10a*, *CsSWEET2a*, *CsSWEET7b*, and *CsSWEET2c*) were up-regulated under polyethylene glycol (PEG) for 72 h. Compared with control, nine genes (*CsSWEET2b*, *CsSWEET5b*, *CsSWEET15c*, *CsSWEET1b*, *CsSWEET9b*, *CsSWEET17a*, *CsSWEET17c*, *CsSWEET7c*, and *CsSWEET15b*) were down-regulated under all PEG treatments (Figure 5). In response to cold, four genes (*CsSWEET3b*, *CsSWEET1a*, *CsSWEET1b*, and *CsSWEET15c*) were up-regulated under all cold treatments. Compared with control, nine genes (*CsSWEET5b*, *CsSWEET2a*, *CsSWEET17b*, *CsSWEET15b*, *CsSWEET17a*, *CsSWEET7a*, *CsSWEET2b*, *CsSWEET3a*, and *CsSWEET17c*) were down-regulated under all cold treatments (Figure 6). Among them, the expression of *CsSWEET1a* was significantly up-regulated in all chosen two treatments, indicating that this gene might help tea resist environmental stresses. This phenomenon was different from previous studies. For example, the banana *MaSWEET1a* was not induced by osmotic, cold, and salt stresses (Miao et al., 2017). The expression level of *GhSWEET1* was only upregulated at 6 h under cold stress, but it was almost not expressed when induced by drought stress (Li et al., 2018). Taken together, we believed that tea plants might have developed specialized regulatory mechanisms for different abiotic stresses.

## Conclusion

In the present study, we identified 28 *CsSWEET*s in the *C. sinensis* genome. Further analyses for ML tree, intron–exon, and duplication suggested that the conservation of *CsSWEET*s was accompanied by a certain degree of divergence. The expression profiles of all *CsSWEET*s suggested that several genes were highly expressed in reproductive tissues, indicating that these genes played important roles in sugar transport. Additionally, 28 *CsSWEET*s were differently expressed for RNA-seq data under cold and drought stresses, indicating that tea plants might have developed specialized regulatory mechanisms for different abiotic stresses.

## Data Availability Statement

The original contributions presented in the study are included in the article/Supplementary Material, further inquiries can be directed to the corresponding author/s.

## Author Contributions

LJ and XZ performed the experiments, analyzed the data, and wrote the manuscript. LJ, CS, XZ, and JY conceived the research and revised the manuscript. All the authors read and approved the final manuscript.

## Conflict of Interest

The authors declare that the research was conducted in the absence of any commercial or financial relationships that could be construed as a potential conflict of interest.

## Footnotes

1 http://tpia.teaplant.org/

2 https://www.arabidopsis.org/

3 https://www.ncbi.nlm.nih.gov/

4 http://pfam.xfam.org

## Supplementary Material

Supplementary Figure 1 The gene structure of *CsSWEET*s in *Camellia sinensis*. Green boxes suggest exons, and gray lines indicate introns.

Supplementary Figure 2 The distribution of conserved motifs in CsSWEETs of *Camellia sinensis*. The MEME was used to identify the motif. The different colors of the boxes indicate different motifs numbered 1–20.

Supplementary Figure 3 Ka/Ks analysis for duplicated *CsSWEET*s paralogs.

Supplementary Table 1 Ka/Ks analysis for duplicated *CsSWEET*s paralogs.
