# Supplementary figures and images for: SWEET Transporters and the Potential Functions of These Sequences in Tea (Camellia sinensis)
Source: Front Genet. 2021 Mar 31;12:655843. doi: 10.3389/fgene.2021.655843 (PMC8044585; doi:10.3389/fgene.2021.655843)

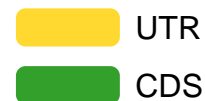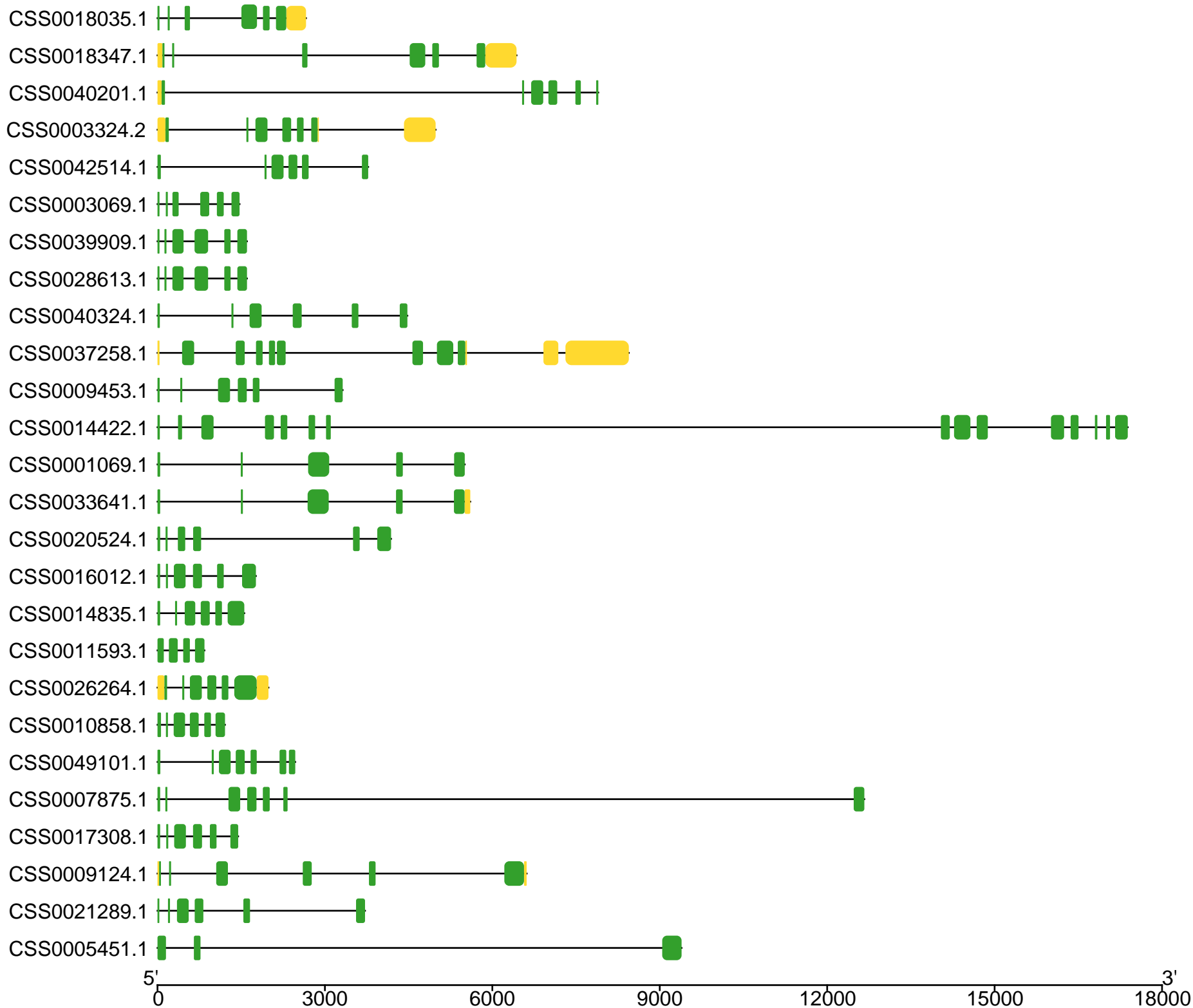

Supplement: Supplementary Figure 1 — The gene structure of CsSWEETs in Camellia sinensis. Green boxes suggest exons, and gray lines indicate introns. [file Image_1.pdf]

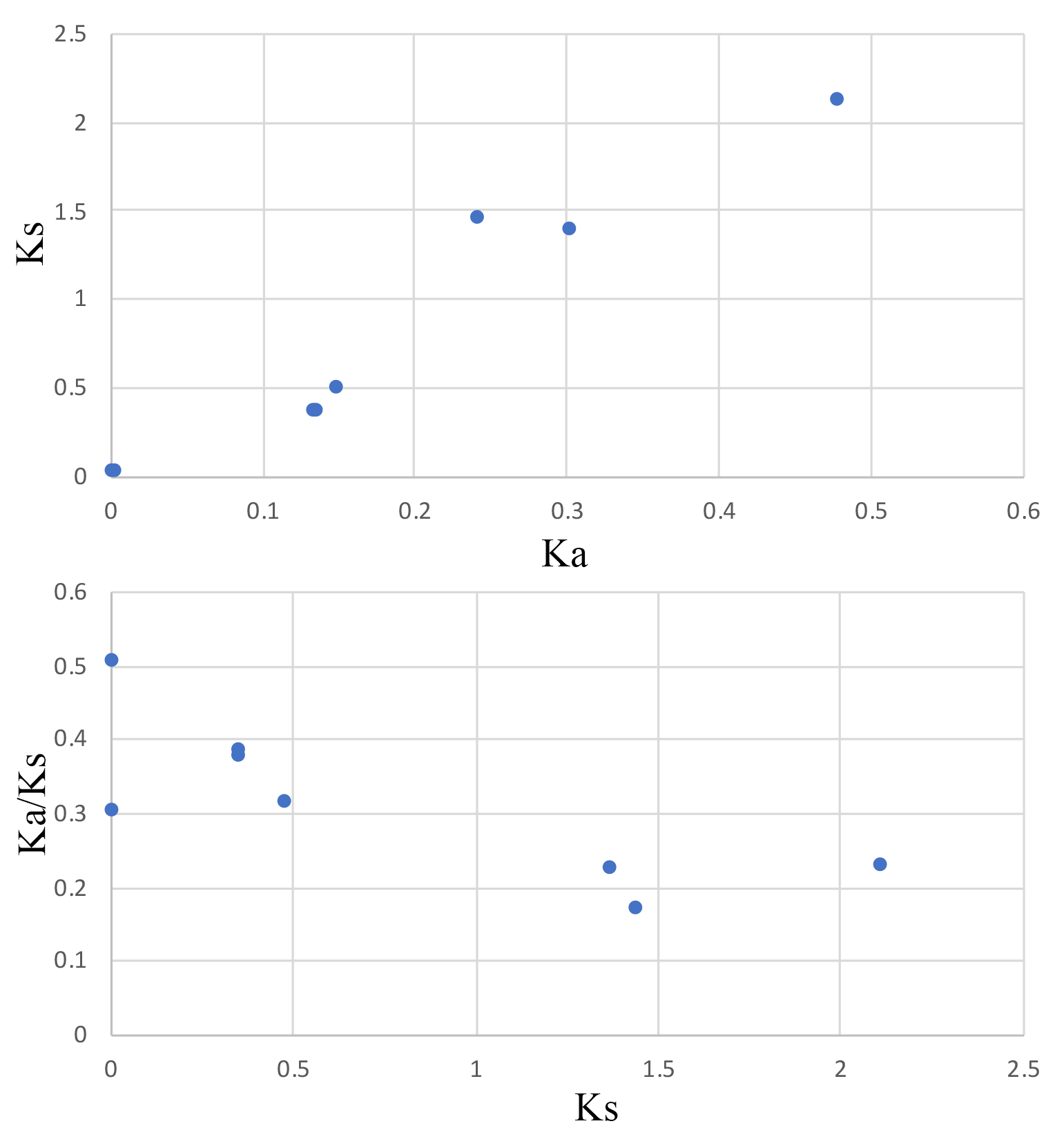

Supplement: Supplementary Figure 3 — Ka/Ks analysis for duplicated CsSWEETs paralogs. [file Image_3.tiff]
