# Supplementary material for: SWEET Transporters and the Potential Functions of These Sequences in Tea (Camellia sinensis)
Source: Front Genet. 2021 Mar 31;12:655843. doi: 10.3389/fgene.2021.655843 (PMC8044585; doi:10.3389/fgene.2021.655843)
Supplement: Supplementary Figure 2 — The distribution of conserved motifs in CsSWEETs of Camellia sinensis. The MEME was used to identify the motif. The different colors of the boxes indicate different motifs numbered 1–20. [file Image_2.pdf]

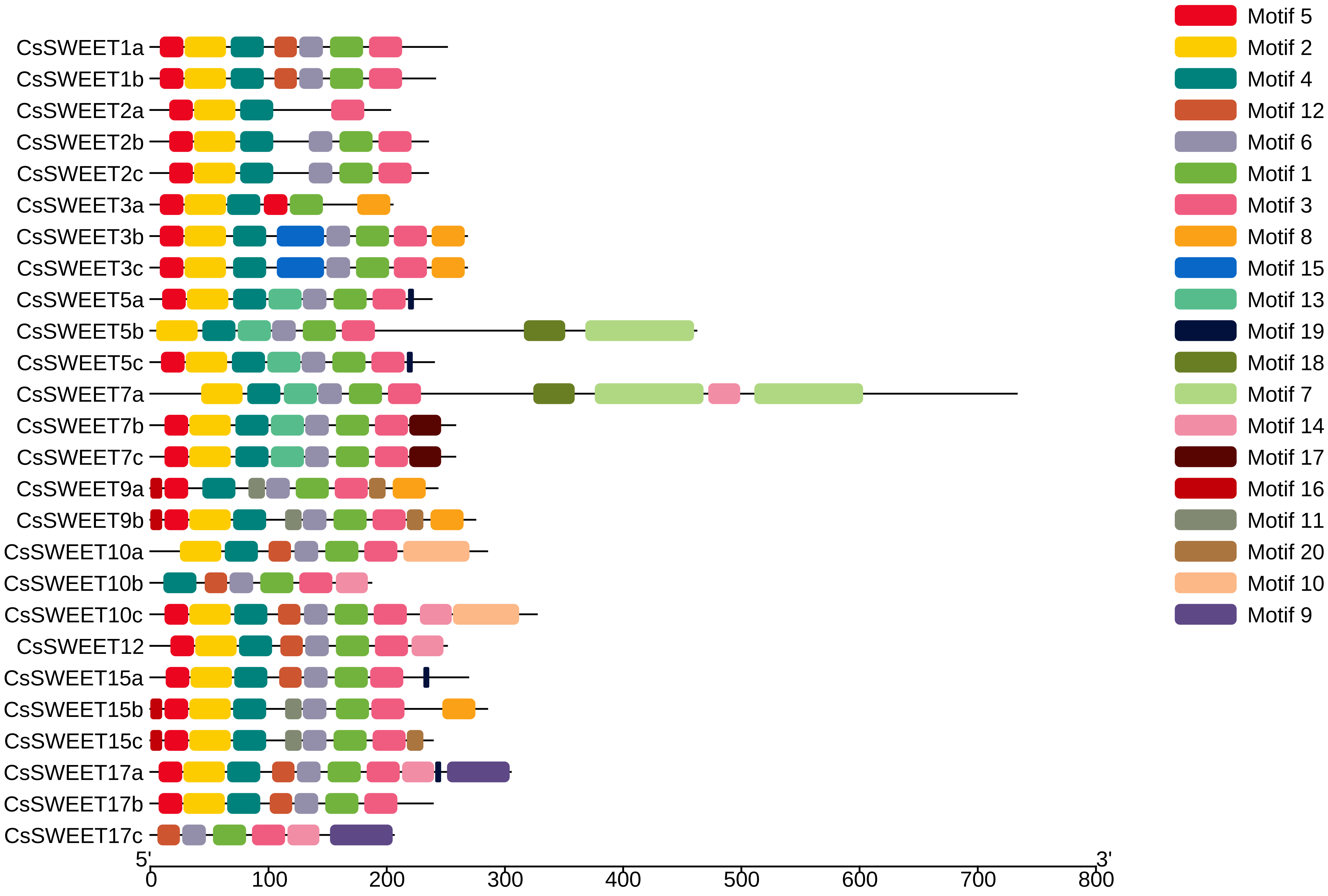

CsSWEET1a
CsSWEET1b
CsSWEET2a
CsSWEET2b
CsSWEET2c
CsSWEET3a
CsSWEET3b
CsSWEET3c
CsSWEET5a
CsSWEET5b
CsSWEET5c
CsSWEET7a
CsSWEET7b
CsSWEET7c
CsSWEET9a
CsSWEET9b
CsSWEET10a
CsSWEET10b
CsSWEET10c
CsSWEET12
CsSWEET15a
CsSWEET15b
CsSWEET15c
CsSWEET17a
CsSWEET17b
CsSWEET17c
5'
3'
0
100
200
300
400
500
600
700
800
Motif 5
Motif 2
Motif 4
Motif 12
Motif 6
Motif 1
Motif 3
Motif 8
Motif 15
Motif 13
Motif 19
Motif 18
Motif 7
Motif 14
Motif 17
Motif 16
Motif 11
Motif 20
Motif 10
Motif 9
